# Improvement of Gaofen-3 Absolute Positioning Accuracy Based on Cross-Calibration

**DOI:** 10.3390/s17122903

**Published:** 2017-12-14

**Authors:** Mingjun Deng, Guo Zhang, Ruishan Zhao, Shaoning Li, Jiansong Li

**Affiliations:** 1 School of Remote Sensing and Information Engineering, Wuhan University, Wuhan 430079, China; dmj2008@whu.edu.cn (M.D.); jiansongli@whu.edu.cn (J.L.); 2 State Key Laboratory of Information Engineering in Surveying, Mapping and Remote Sensing, Wuhan University, Wuhan 430079, China; shaoningli@whu.edu.cn; 3 School of Geomatics, Liaoning Technical University, Fuxin 123000, China; zhaoruishan333@163.com

**Keywords:** Gaofen-3, geometric accuracy, cross-calibration

## Abstract

The Chinese Gaofen-3 (GF-3) mission was launched in August 2016, equipped with a full polarimetric synthetic aperture radar (SAR) sensor in the C-band, with a resolution of up to 1 m. The absolute positioning accuracy of GF-3 is of great importance, and in-orbit geometric calibration is a key technology for improving absolute positioning accuracy. Conventional geometric calibration is used to accurately calibrate the geometric calibration parameters of the image (internal delay and azimuth shifts) using high-precision ground control data, which are highly dependent on the control data of the calibration field, but it remains costly and labor-intensive to monitor changes in GF-3’s geometric calibration parameters. Based on the positioning consistency constraint of the conjugate points, this study presents a geometric cross-calibration method for the rapid and accurate calibration of GF-3. The proposed method can accurately calibrate geometric calibration parameters without using corner reflectors and high-precision digital elevation models, thus improving absolute positioning accuracy of the GF-3 image. GF-3 images from multiple regions were collected to verify the absolute positioning accuracy after cross-calibration. The results show that this method can achieve a calibration accuracy as high as that achieved by the conventional field calibration method.

## 1. Introduction

Gaofen-3 (GF-3) is one of the most important satellites in the China Earth Observation System. With high resolution, a large imaging swath, and the ability to image on both left and right sides, GF-3 can monitor global land and ocean at any time of the day or night under all weather conditions and provide stable, high-quality observation data [1]. The GF-3 mission was launched in August 2016, equipped with a full polarimetric synthetic aperture radar (SAR) sensor in the C-band. The GF-3 has 12 imaging modes, such as stripmap, sliding-spot, and scansar, and is the SAR satellite with the most imaging modes. The spatial resolution varies from 1 to 500 m and the swath varies from 10 to 650 km. In addition, GF-3 is the first low-orbit remote sensing satellite with a long-life design in China. The design life of 8 years is longer than both the 3~5-year life cycle of remote sensing satellites and the 6~7-year life cycle of international remote sensing satellites [2].

In recent years, the rapid global development of space-borne SAR has led to the continuous improvement of image product quality, such as the European Remote Sensing (ERS) Satellite ERS-1/2 [3], the Japanese Advanced Land Observing Satellite (ALOS-PALSAR) launched in 2006 [4], the Italian Constellation of Small Satellites for Mediterranean basin Observation (COSMO-SkyMed) series SAR launched in 2007 and 2008 [5], the Canadian RadarSat-2 [6,7], the German TerraSAR-X [8,9], and the European Space Agency sentinel series [10,11]. At present, the absolute positioning accuracy of these satellites can reach less than 10 m, especially TerraSAR-X products, in which absolute positioning accuracy on the decimeter level can be achieved after geometric calibration [12].

Geometric calibration is crucial for improving the absolute positioning accuracy of satellite images through the use of ground control data to precisely calibrate the geometric calibration parameters (internal delay and azimuth shifts) of satellites. Therefore, after the satellite has been launched, it is necessary to collect images of the calibration field to complete the geometric calibration. At present, both domestic and international research on the geometric calibration of SAR satellites is advanced and fully verified for ALOS-PALSAR, TerraSAR-X, Sentinal-1A, YaoGan-13A (YG-13A) [13], and other high-resolution SAR satellites. After a systematic review of the existing literature [2,3,4,5,6,7,8,9,10,11,12,13], we found that the conventional geometric calibration method requires ground control data of the calibration field, which exposes the following problems for practical applications.

**Figure 1:**
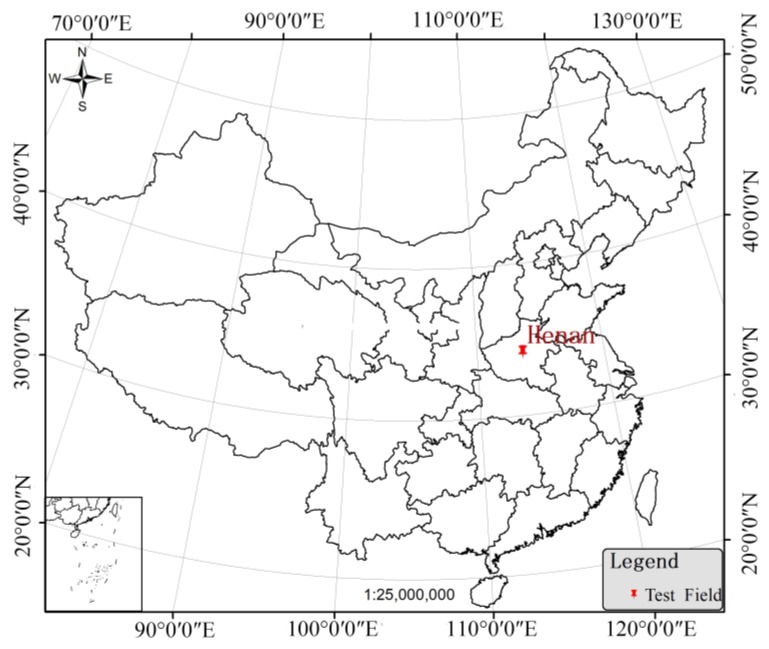
Location of the one specific synthetic aperture radar geometric calibration field in Henan province, China.

Absolute positioning accuracy may change with increased satellite running time. Regular geometric calibration of satellites and monitoring of the changes in the geometric calibration parameters are helpful to grasp the degradation mechanism of SAR satellite absolute positioning accuracy and to provide references for satellite design. However, geometric calibration parameters can only be calibrated when the satellite successfully obtains the calibration field image. There is only one specific SAR geometric calibration field in China, located in Henan province (Figure 1). Moreover, the temporary geometric calibration field must be artificially pre-laid with corner reflectors, the cost of which is high. These factors lead to a low calibration data acquisition frequency of once a year and sometimes less frequent. Therefore, it is difficult to use conventional field calibration methods to monitor changes in the geometric calibration parameters for the satellite.

For optical satellite geometric calibration, it is necessary to obtain high-precision digital elevation models (DEMs) and digital orthophoto models (DOMs) for the calibration field [14]. In theory, this method can also be applied to space-borne SAR satellite geometric calibration, obtaining control points from DOMs and DEMs. However, high-precision DOM and DEM data are often obtained by aerial photogrammetry, and the acquisition cost is high. Because of rapid changes of ground objects, DOMs and DEMs cannot be updated in time, which leads to the difficulty of selecting control points.

However, the absolute positioning accuracy of space-borne SAR images is improving. For example, without considering elevation errors, the absolute positioning accuracy of YG-13A in stripmap mode is higher than 3 m after calibration, and that in sliding spotlight mode is higher than 1.5 m [13]. The YG-13A mission was launched in late 2015. YG-13A has subsequently acquired images from many parts of the world, and these images can be used for plane positioning control data. In this study, we propose geometric cross-calibration for improving the absolute positioning accuracy of GF-3. This method first extracts conjugate points from YG-13A and GF-3 images under the same incidence angle. It then recovers precise GF-3 geolocation parameters based on the geometric restriction that the conjugate points should be positioned at the same location; there is no need to use high-precision DEM data in the calibration process. It therefore solves the problem of the conventional geometric calibration method related to its dependence on ground control data of the calibration field, i.e., the geometric calibration can be completed as long as an image pair with a similar incidence angle is obtained. The absolute positioning accuracy of GF-3 after cross-calibration is validated using GF-3 images over several test sites. By comparing the absolute positioning accuracy of GF-3 after cross-calibration with that after the conventional calibration method, we validated the effectiveness and feasibility of the proposed method.

## 2. Error Sources of GF-3 Absolute Positioning

SAR, as a kind of active remote sensor, can provide a precise distance between the sensor and target and the Doppler frequency of the echo wave. In the imagery process, the absolute location of the image pixel can be determined by these two factors. In Equation (1), the image pixel coordinates (*i,j*) and the target ground position (*x_t_,y_t_,z_t_*) can be accurately correlated using the range-Doppler model [15]:

(1) $$\left\{ \begin{array}{l} \left|\vec{R_{s}}-\vec{R_{t}}\right|=R\\ f_{d}=-\frac{2}{\lambda R}\left(\vec{V_{s}}-\vec{V_{t}}\right)\cdot (\vec{R_{s}}-\vec{R_{t}})\\ \frac{x_{t}{}^{2}+y_{t}{}^{2}}{{\left(R_{e}+h_{t}\right)}^{2}}+\frac{z_{t}{}^{2}}{R_{p}{}^{2}}=1 \end{array}\right.,$$

where $\vec{R_{t}}=[x_{t},y_{t},z_{t}{]}^{T}$ is the SAR ground target vector; $\vec{R_{s}}=[x_{s},y_{s},z_{s}{]}^{T}$ is the position vector of the phase center of the SAR antenna; *R* is the slant range between the target and the satellite; *f_d_* is the Doppler center frequency for SAR imaging; *λ* is the radar wavelength of SAR; $\vec{V_{s}}=[v_{s_{x}},v_{s_{y}},v_{s_{z}}{]}^{T}$ is the velocity vector of the phase center of the SAR antenna; $\vec{V_{t}}=[v_{t_{x}},v_{t_{y}},v_{t_{z}}{]}^{T}$ is the velocity vector of the target T; *R_e_* = 6378.139 is the semi-major axis of the WGS-84 ellipsoid; *h_t_* is the height of the target relative to the surface of the Earth; and *R_p_*, the semi-minor axis of the WGS-84 ellipsoid, is given by:

(2) $$R_{p}=(1-f)(R_{e}+h_{t}),$$

where *f* is the flattening factor and *f* = 1/298.255. 

In Equation (1), the slant range *R* between the target and the satellite is given by:

(3) $$R=R_{near}+i\frac{c}{2f_{s}},$$

where *R_near_* is the slant-range of the first range gate which is determined by the radar pulse propagation time; *i* is the range pixel coordinate of the target in the image; *c* is the propagation velocity of microwaves in the atmosphere; and *f_s_* is the sampling frequency of the pulse.

The position and velocity vector of the phase center of the SAR antenna are calculated using the Lagrange polynomial insert according to the imaging time of the target $\eta_{p}$; $\eta_{p}$ is defined by:

(4) $$\eta_{p}=\eta_{0}+\frac{j}{PRF},$$

where $\eta_{0}$ is the azimuth time of the first image line; *j* is the azimuth pixel coordinate of the point target in the image; and *PRF* is SAR pulse repetition frequency.

The process of solving the range-Doppler model is identical to the process of the absolute pixel location of the SAR image. Given the row and column index (*i,j*) of a pixel in the image, the longitude and latitude of the pixel can be calculated according to the range-Doppler model. The absolute positioning accuracy of space-borne SAR is mainly affected by satellite position and velocity error, SAR system time error, atmospheric propagation delay error, processor-induced errors, and terrain height error, described in the following subsections [2].

### 2.1. Satellite Position and Velocity Error

In order to locate SAR image objects in a reference frame, the position and velocity of the SAR antenna phase center in space are required. GF-3 uses dual frequency GPS receiver for orbit determination. After precise track processing, the position and velocity accuracy can reach 5 cm and 0.05 mm/s, respectively [2]. The residual satellite position and velocity error are random errors and cannot be eliminated by ground treatment methods. This affects the theoretical limit of the absolute positioning accuracy of GF-3.

### 2.2. SAR System Time Error

The SAR system time error mainly refers to the azimuth time synchronization error and the internal electronic delay of the instrument. The azimuth time synchronization error is mainly caused by the fixed deviation between the satellite local clock and the navigation timing system, which mainly results in the azimuth positioning error. The internal electronic delay of the instrument will lead to the range positioning error. The SAR system time error is the main error source for the absolute positioning of space-borne SAR. For the same imaging mode, the error value is relatively stable and can be calculated by the method of geometric calibration [16,17]. 

### 2.3. Atmospheric Propagation Delay Error

Due to the existence of the atmospheric path delay, the SAR slant range has several meters of measurement error. The atmospheric propagation delay of radar signals is mainly related to atmospheric pressure intensity, temperature, water vapor content, ionospheric electron density, and the emission frequency of radar signals. Therefore, the atmospheric propagation delay error is a systematic error related to the incidence angle of the radar beam and the imaging time of the SAR image. The effect can be modeled and eliminated [18,19]. Typically, the atmospheric path delay correction model is the product of the mapping function and the atmospheric zenith delay [20]:

(5) $$\delta_{atmo}=\Delta L_{z}\times m(\theta ),$$

where $\Delta L_{z}$ is the atmospheric zenith delay and the mapping function *m*(*θ*) = 1/cos(*θ*); and *θ* denotes the incidence angle.

### 2.4. Processor-Induced Errors

SAR imaging processing is usually based on a “stop-and-go” approximation model. It is assumed that the SAR satellite is stationary during the transmission of the pulse until completion of the reception of the pulse and then moves to the next position for the transmission and reception of the next pulse. However, in reality, the satellite moves a certain distance along its orbit during the time between pulse transmission and echo reception. This is referred to as the “bistatic effect” or “stop-and-go approximation” and has been studied for many years [10,21]. In the process of GF-3 absolute positioning, the range-Doppler equations for the real continuously moving configuration is established to eliminate this error [22].

### 2.5. Terrain Height Error

**Figure 2:**
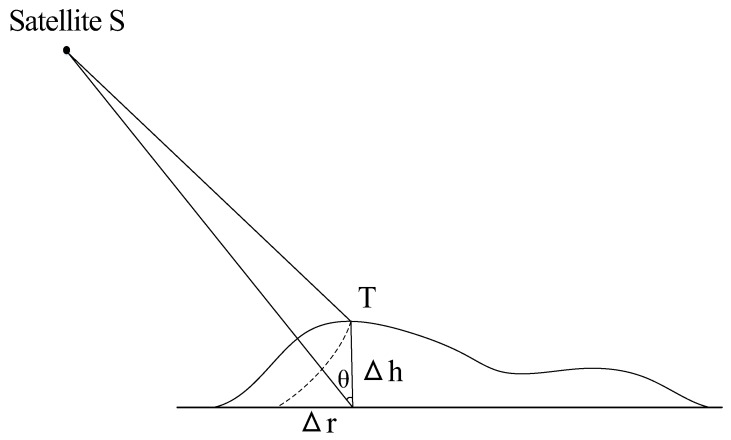
Positioning error caused by terrain height error.

Because SAR is a side-looking imaging apparatus and can only measure distance and Doppler two-dimensional information, accurate positioning depends on the support of external elevation data. As shown in Equation (6) and Figure 2, a terrain height error of Δ*h* will result in a horizontal positioning error of Δ*r*:

(6) $$\Delta r=\frac{\Delta h}{\tan(\theta )},$$

where *θ* is the incidence angle.

## 3. Geometric Calibration

Due to the above errors, there is a deviation in the absolute positioning of SAR. In practice, the control point can be used to improve the positioning accuracy of each scene image, but this method requires many control points [23,24]. Through the analysis of the previous section, we know that the azimuth time synchronization error and the internal electronic delay of the instrument that affect the positioning accuracy are relatively stable and will not change over time. Therefore, to improve the absolute positioning accuracy, we can calibrate the geometric calibration parameters of the image (internal delay and azimuth shifts) using high-precision ground control data. The classical geometric calibration schemes all take into account the ascending and descending modes, left and right side looks, and different beam positions. However, these considerations are not the main factors that affect the calibration parameters of the physical properties of the SAR signal. In addition, the current spaceborne SAR systems have dozens or even hundreds of beam positions. It is not realistic to calibrate every beam position. Take into account the cause of the positioning error, it is reasonable to adopt different imaging mode schemes for high-precision geometric calibration [13].

### 3.1. Conventional Field Calibration

It is assumed that the system azimuth time synchronization error is $\Delta t_{a}$ and the system internal delay is $\Delta t_{r}$. In addition, considering the atmospheric delay $\delta_{delay}$, Equations (3) and (4) can be written as Equation (7):

(7) $$\left\{ \begin{array}{l} R=R_{near}+\frac{\Delta t_{r}\times c}{2}+\delta_{delay}+i\frac{c}{2f_{s}}\\ \eta_{p}=\eta_{0}+\Delta t_{a}+\frac{j}{PRF} \end{array}\right.,$$

The geometric calibration model for space-borne SAR can be expressed as follows [25]:

(8) $$\left\{ \begin{array}{l} F_{i}=R-[R_{near}+\frac{\Delta t_{r}\times c}{2}+\delta_{delay}+i\frac{c}{2f_{s}}]=0\\ F_{j}=\eta_{p}-[\eta_{0}+\Delta t_{a}+\frac{j}{PRF}]=0 \end{array}\right.,$$

The error equation for Equation (8) is as follows:

(9) V=Bx−L,

where $B=\left[\begin{array}{l} \frac{\partial F_{i}}{\partial \Delta t_{r}}\frac{\partial F_{i}}{\partial \Delta t_{a}}\\ \frac{\partial F_{j}}{\partial \Delta t_{r}}\frac{\partial F_{j}}{\partial \Delta t_{a}} \end{array}\right]$; $x={\left[d\Delta t_{r},d\Delta t_{a}\right]}^{T}$; and $L=\left[\begin{array}{l} -F_{i}^{0}\\ -F_{j}^{0} \end{array}\right]$. $F_{i}{}^{0}$ and $F_{j}{}^{0}$ mean the deviation between the observed value and the calculated value of range and azimuth pixel coordinate of the target in the image, respectively.

The steps of conventional field calibration are as follows:

1. Mount the corner reflectors in the calibration field area, obtain ground positions (*x_t_,y_t_,z_t_*) of the corner reflectors, and acquire calibration field images.
2. Extract the accurate image coordinates (*i,j*) of the corner reflectors, and calculate *R* and *η_p_* and by using the inverse location algorithm [26] according to Equation (1).
3. Calculate the atmospheric propagation delay, and calculate the correction values for the atmospheric propagation delay $\delta_{delay}$ according to the National Centers for Environmental Prediction global atmospheric parameters, updated every 6 h, and the global vertical total electron content data provided by the Center for Orbit Determination in Europe.
4. Obtain accurate values of ${\left[\Delta t_{r},\Delta t_{a}\right]}^{T}$ by applying Equations (7) and (8).

### 3.2. Cross-Calibration

**Figure 3:**
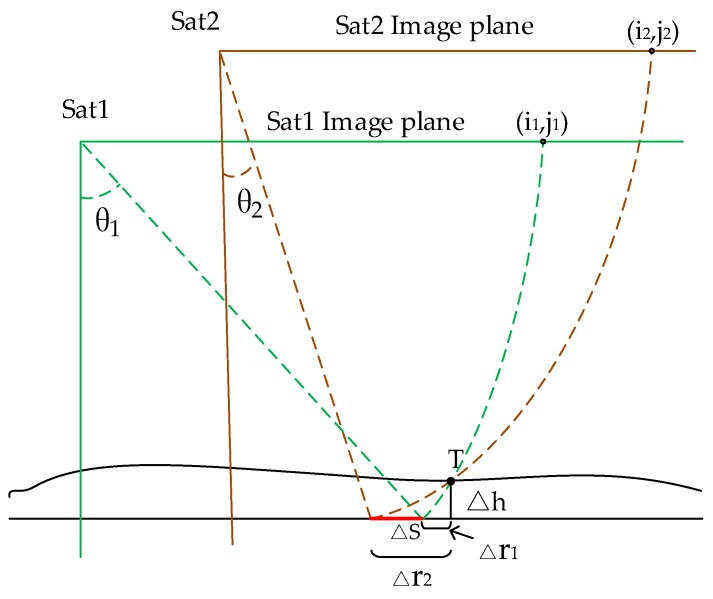
Schematic diagram showing the intersection of conjugate points (*i*_1_*,j*_1_) and (*i*_2_*,j*_2_). Δ*h*: elevation error of T; △r_1_ and △r_2_: positioning errors caused by elevation error; Δ*S*: deviation of ground plane; Sat 1 and Sat2: satellites; T: ground point; *θ*_1_ and *θ*_2_: incidence angles.

As shown in Figure 3, satellites Sat1 and Sat2 imaged the same ground point, T, at (*i*_1_*,j*_1_) and (*i*_2_*,j*_2_) on the Sat1 and Sat2 image plane, respectively. 

Assuming that the geolocation parameters of Sat1 and Sat2 (including measuring track, slant range, and Doppler parameters) are accurate and the elevation of T is correct according to the geometric positioning model, the conjugate points (*i*_1_*,j*_1_) and (*i*_2_*,j*_2_) should be positioned at the same location T by an indirect localization algorithm of the range-Doppler location model. However, it is often difficult to locate (*i*_1_*,j*_1_) and (*i*_2_*,j*_2_) at the same point on the ground when utilizing satellite data. This is caused by the geolocation error and stereoscopy errors induced by the elevation error of the ground object T. 

Based on the features of SAR side-look imaging, the deviation Δ*S* in Figure 3 caused by the elevation error can be calculated as follows:

(10) $$\Delta S=\Delta h/\tan(\theta_{2})-\Delta h/\tan(\theta_{1}),$$

where *θ*_1_ and *θ*_2_ denote the incidence angles of (*i*_1_*,j*_1_) and (*i*_2_*,j*_2_), respectively; and Δ*h* is the elevation error of T, which depends on the topographic data adopted for geometric positioning (e.g., global open Shuttle Radar Topography Mission (SRTM) data). Based on Equation (10), when *θ*_1_ and *θ*_2_ are close enough (i.e., the two satellites scan one region with similar incidence angles), the deviation Δ*S* caused by the elevation error, can be neglected. In that case, Δ*S* is only caused by the geolocation parameter error and can be calculated using the following equation:

(11) $$\Delta S=f_{sat1}(i1,j1)-f_{sat2}(i2,j2),$$

where *f_sat_*_1_ and *f_sat_*_2_ denote the geolocation parameter errors of Sat1 and Sat2, respectively. If the geolocation accuracy of Sat1 is very accurate (i.e., *f_sat_*_1_(*i*_1_*,j*_1_) = 0), Equation (11) can be written as follows:

(12) $$\Delta S=-f_{sat2}(i2,j2),$$

**Figure 4:**
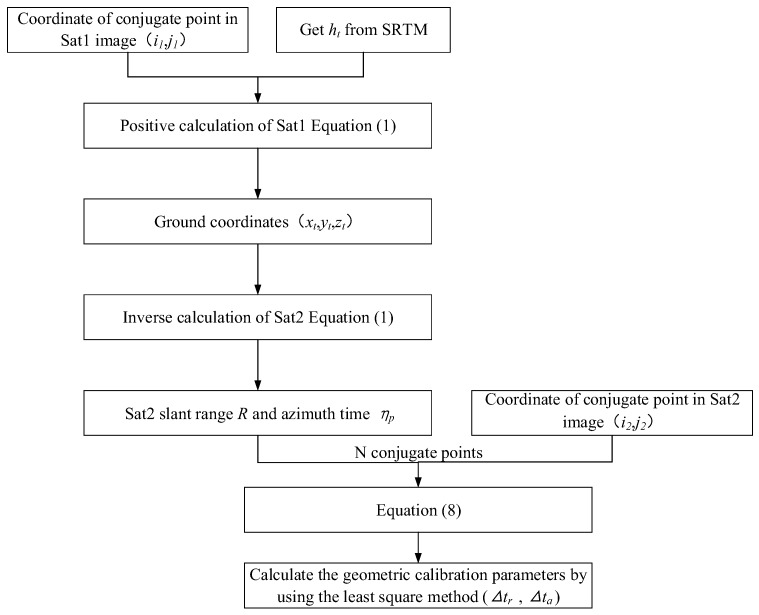
Flow chart of cross-calibration algorithm; *h_t_*; height of the target relative to the surface of the Earth; SRTM: Shuttle Radar Topography Mission.

As shown in Figure 4, assuming that the geolocation accuracy of Sat1 is very accurate, the conjugate points (*i*_1_*,j*_1_) and (*i*_2_*,j*_2_) can be acquired by matching Sat1 and Sat2 satellite images and calculating the ground coordinates (*x_t_,y_t_,z_t_*) corresponding to (*i*_1_*,j*_1_) in Sat1, using the Sat1 range-Doppler model and the SRTM-DEM. 

Subsequently, (*x_t_,y_t_,z_t_*) can be substituted into the Sat2 range-Doppler model, and *R* and *η_p_* can be obtained when the ground target is imaged by Sat2. Thereafter, *R*, *η_p_* and (*i*_2_*,j*_2_) can be substituted into the geometric calibration model (Equation (8)), and the internal electronic delay of the instrument (Δ*t_r_*) and the systematic azimuth shifts (Δ*t_a_*) of Sat2 can be calculated.

From the above analysis, it is clear that the cross-calibration method is consistent with the conventional field calibration. The difference is that the control point acquisition method used to solve the calibration equation is not the same. The conventional field calibration uses the ground coordinates of corner reflectors (*x_t_,y_t_,z_t_*) and their image plane coordinates (*i,j*) to solve Equation (8). The cross-calibration method only needs a cross-calibration image pair with similar incidence angles, as the reference image of the cross-calibration image pair provides high-precision plane control data. Furthermore, the requirement of high-precision DEM data is eliminated by the limited condition of similar incidence angles for the cross-calibration image pair. When the reference data is well calibrated, the absolute error of the image to be calibrated can be obtained. Otherwise, only the relative error can be obtained.

## 4. Experiment and Analysis

### 4.1. Experimental Data

Three datasets (Data A, B, and C) were adopted for validating the proposed method. Data A is the YG-13A image with stripmap mode, while Data B and C were images of GF-3 with fine stripmap 1 mode. To summarize, Data A and B formed the cross-calibration image pair for the geometric calibration of GF-3. Data C was used to validate the absolute positioning accuracy of GF-3 after cross-calibration and conventional field calibration, respectively. Table 1 lists the experimental image specifications.

13-HN-2016-03-11 from Data A and GF3-HN-2016-11-29 from Data B are YG-13A and GF-3 images of the same area, respectively, with relatively similar incidence angles. Based on the incidence angles of 13-HN-2016-03-11 and GF3-HN-2016-11-29 shown in Table 1, the intersection deviation caused by the elevation error can be calculated as follows:

(13) $$\Delta S=\frac{\Delta h}{\tan(37.43)}-\frac{\Delta h}{\tan(37.21)}=-0.010\Delta h,$$

The 90 m SRTM data, with an accuracy of higher than 30 m, may cause an intersection deviation of 0.14 pixels in the GF-3 image. Therefore, the height error of the 90 m SRTM data can be neglected in geometric cross-calibration, and 13-HN-2016-03-11 from Data A and GF3-HN-2016-11-29 from Data B can be adopted for geometric cross-calibration. In general, an intersection deviation of less than 0.2 pixels is tolerable. Considering the use of SRTM as the source of elevation data, Table 2 shows the maximum incidence angle differences under different image resolutions and incidence angles. The maximum incidence angle difference increases with increased image resolution and incidence angle. However, excessive incidence angles will lead to image distortion, and the choice of incidence angle therefore needs to be moderate.

**Figure 5:**
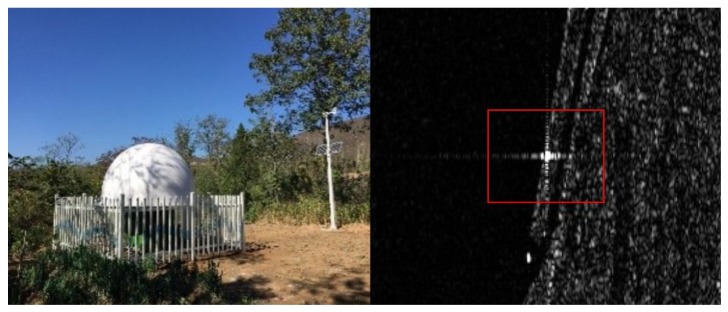
Appearance of the corner reflector (**left**) and its image performance (**right**).

GF-3 images in Data C were used to validate the absolute positioning accuracy of GF-3 after cross-calibration; the corner reflectors (Figure 5), the 1:5000 scale DOM and DEM of the Taiyuan region, and the 1:2000 scale DOM and DEM of the Tianjin region were used as control data to obtain checkpoints to validate the absolute positioning accuracy after calibration. Natural targets in the Taiyuan and Tianjin regions, such as road intersections, water bodies, or field boundaries, were used throughout to serve as checkpoints. The latitude and longitude of checkpoints were obtained from the DOM, and their elevation was obtained from the DEM. Their planimetric accuracies were <1 m, while their height accuracies were <2 m. Through the range-Doppler positioning equation, the image coordinates of the checkpoints were predicted and compared with the measured image coordinates of checkpoints. The absolute location error was obtained as Equation (14); the calculation of absolute location error is described in detail in the literature [10].

(14) $$\left\{ \begin{array}{l} ALE_{\mathrm{rg}}=\;\mathrm{predicted}\;\mathrm{range}\;\mathrm{sample}-\mathrm{measured}\;\mathrm{range}\;\mathrm{sample}\\ ALE_{\mathrm{az}}=\;\mathrm{predicted}\;\mathrm{azimuth}\;\mathrm{sample}-\mathrm{measured}\;\mathrm{azimuth}\;\mathrm{sample} \end{array}\right.,$$

### 4.2. Cross-Calibration Results

**Figure 6:**
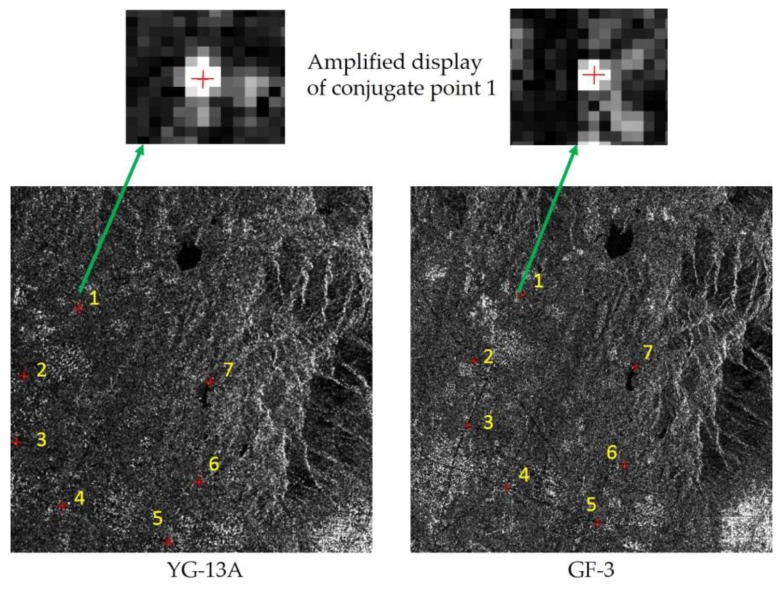
Distribution of conjugate points for cross-calibration image pair from the YG-13A (**left**) and Gaofen-3 satellites (**right**).

Firstly, the corner reflectors were used as checkpoints to validate the absolute positioning accuracy of the reference image in the cross-calibration image pair. The absolute positioning accuracy of 13-HN-2016-03-11 for the range and azimuth was 0.14 m and 0.27 m, respectively. As shown in Figure 6 seven conjugate points were manually extracted from cross-calibration image pair (13-HN-2016-03-11 for Data A and GF3-HN-2016-11-29 for Data B). A problem in the acquisition of conjugate points was the pixel mismatch. As the incidence angles were close, the deformation information of the image pair was almost the same. The conjugate point uncertainty in the SAR images could be estimated to be in the order of one pixel for such features (Figure 6).

The 90 m SRTM was adopted for cross-calibration. Table 3 lists the geometric calibration parameters of GF-3 solved by cross-calibration. There was a fixed deviation in the azimuth and range of the GF-3 image.

The geometric calibration parameters were used to compensate for Data C, and the absolute positioning accuracy of Data C was validated by using checkpoints. Geolocation errors in the image space performed in two directions: azimuth and range. In Table 4 presents statistics on the experimental results.

As can be seen from Table 4, the root mean square error (RMSE) of the range offset varied between 1.17 m and 3.56 m, whereas the mean value across all test images was 2.39 m. In the process of geometric calibration, the instrument delay of the SAR instrument and an additional delay due to the atmospheric propagation path (troposphere and ionosphere) were considered. The remaining uncertainty of pixel localization accuracy for the range was 2.39 m (approximately 1 pixel) after applying all previously described corrections. Pixel localization for the azimuth was slightly better than that for the range; the mean value across all test images was 1.18 m (approximately 0.5 pixels). This shows good geolocation accuracy of the images after calibration.

### 4.3. Comparison with Conventional Field Calibration

**Figure 7:**
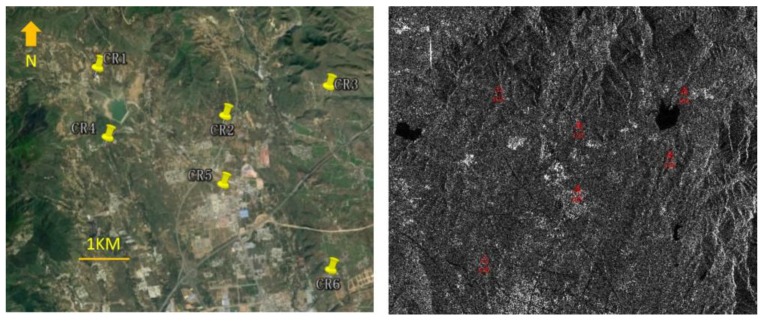
Distribution of six corner reflectors (CR) in the Google Earth (**left**) and Data B image (**right**).

Six corner reflectors were mounted in the Data B coverage area, as shown in Figure 7. We used these control points to complete the conventional field calibration for GF-3. Table 5 lists the geometric calibration parameters of GF-3 solved by conventional field calibration. As mentioned before, Data C was used to verify the absolute positioning accuracy after conventional field calibration.

Table 6 shows the statistical results of the GF-3 absolute positioning accuracy after conventional field calibration. The mean values of RMSEs across all test images were 2.30 m for the range and 0.94 m for the azimuth. Statistical results comparing the absolute positioning accuracy of GF-3 after conventional field calibration with that after cross-calibration are shown in Table 7. The results show that the accuracy achieved by the cross-calibration method was as high as that achieved by the conventional field calibration method. In contrast to the conventional field calibration method, the accuracy of the cross-calibration method was mainly affected by the absolute positioning accuracy of the reference image and the matching accuracy of the cross-calibration image pair. The absolute positioning accuracy of the YG-13A image was high and can be used as reference image. Cross-calibration requires the incidence angles of the two images to be similar, which reduces image deformation to some extent and ensures matching accuracy.

## 5. Conclusions

Geometric calibration is a key technology for improving the absolute positioning accuracy of Gaofen-3 (GF-3). Conventional field calibration remains the most widely used method of synthetic aperture radar (SAR) geometric calibration, but due to its high cost, researchers have been searching for an alternative method. The results of this paper show that the cross-calibration method has great potential. No high-precision digital elevation model (DEM) data is required, and the accuracy of the calibration is comparable to that of conventional calibration. However, cross-calibration has some restrictions, e.g., the incidence angles of the cross image pair should be approximately the same. In addition, the difference in the resolution of the two images in an image pair cannot be too large, otherwise it will affect the extraction accuracy of the conjugate points. To overcome the limitation posed by the need for similar incidence angles is scope for further research.

## Figures and Tables

**Table 1:** Experimental image specifications for validation of the proposed method.

Satellite	ID	Pixel Distance (m)		Imaging Time	Incidence Angle (°)	Orbit	Side
		Range	Azimuth				
Data A							
YG-13A	13-HN-2016-03-11	0.6	0.9	11 March 2016	37.21	Desc	R
Data B							
GF-3	GF3-HN-2016-11-29	2.2	2.8	14 October 2016	37.43	Desc	R
Data C							
GF-3	GF3-HN-2016-12-30	2.2	2.6	30 December 2016	38.66	Asc	R
GF-3	GF3-TJ-2017-02-17	2.2	3.1	17 February 2017	33.82	Desc	R
GF-3	GF3-TJ-2017-03-18	2.2	3.1	18 March 2017	33.82	Desc	R
GF-3	GF3-TY-2016-12-30	2.2	2.6	30 December 2016	38.66	Asc	R
GF-3	GF3-TY-2017-01-11	2.2	2.8	11 January 2017	40.07	Asc	R

Desc = Descending; Asc = Ascending; L = Left; R = Right.

**Table 2:** Maximum incidence angle differences under different image resolutions and different incidence angles.

Image Resolution (m)	1	2	4	6	8	10
Incidence Angle (°)						
20	0.040°	0.085°	0.175°	0.260°	0.350°	0.435°
30	0.095°	0.185°	0.375°	0.560°	0.745°	0.925°
40	0.155°	0.310°	0.620°	0.925°	1.225°	1.525°
50	0.220°	0.440°	0.885°	1.315°	1.745°	2.165°
60	0.285°	0.565°	1.130°	1.685°	2.235°	2.780°

**Table 3:** Geometric calibration parameters of Gaofen-3 solved by cross-calibration.

Direction	Item	Value
Azimuth	$\Delta t_{a}$	+0.322 ms
Range	$\Delta t_{r}$	−61.02 ns

**Table 4:** Absolute positioning accuracy after cross-calibration. RMSE: root mean square error.

Test Site	ID	Range (m)			Azimuth (m)		
		Max	Min	RMSE	Max	Min	RMSE
Songshan	GF3-HN2016-12-30	4.21	3.13	3.56	−2.09	−0.98	1.58
Tianjin	GF3-TJ-2017-02-17	−3.50	−0.79	2.20	−2.21	−0.31	1.53
Tianjin	GF3-TJ-2017-03-18	−1.86	0.48	1.17	−1.66	−0.56	1.00
Taiyuan	GF3-TY-2016-12-30	3.80	0.62	2.73	−1.77	−0.08	0.97
Taiyuan	GF3-TY-2017-01-11	2.49	1.82	2.26	−0.93	−0.05	0.83
Average		-	-	2.39	-	-	1.18

**Table 5:** Geometric calibration parameters of Gaofen-3 solved by conventional field calibration.

Direction	Item	Value
Azimuth	$\Delta t_{a}$	+0.371 ms
Range	$\Delta t_{r}$	−61.95 ns

**Table 6:** Absolute positioning accuracy after conventional field calibration. RMSE: root mean square error.

Test Site	ID	Range (m)			Azimuth (m)		
		Max	Min	RMSE	Max	Min	RMSE
Songshan	GF3-HN2016-12-30	3.93	2.86	3.28	−1.77	−0.65	1.28
Tianjin	GF3-TJ-2017-02-17	−3.78	−1.07	2.46	−1.88	0.02	1.24
Tianjin	GF3-TJ-2017-03-18	−2.14	0.20	1.31	−1.33	−0.23	0.71
Taiyuan	GF3-TY-2016-12-30	3.53	0.34	2.47	−1.44	−0.13	0.76
Taiyuan	GF3-TY-2017-01-11	2.21	1.54	1.98	1.22	−0.02	0.73
Average		-	-	2.30	-	-	0.94

**Table 7:** Comparison between conventional field calibration (A) and cross-calibration (B) methods.

Test Site	ID	Range (m)		Azimuth (m)	
		A	B	A	B
Songshan	GF3-HN2016-12-30	3.28	3.56	1.28	1.58
Tianjin	GF3-TJ-2017-02-17	2.46	2.2	1.24	1.53
Tianjin	GF3-TJ-2017-03-18	1.31	1.17	0.71	1.00
Taiyuan	GF3-TY-2016-12-30	2.47	2.73	0.76	0.97
Taiyuan	GF3-TY-2017-01-11	1.98	2.26	0.73	0.83
Average		2.30	2.39	0.94	1.18

## References

[1] Zhang Q. (2017). System Design and Key Technologies of the GF-3 Satellite. Acta Geod. Cartogr. Sin..

[2] Ding C., Liu J., Lei B. (2017). Preliminary exploration of systematic geolocation accuracy of GF-3 SAR satellite system. J. Radars.

[3] Mohr J.J., Madsen S.N. (2001). Geometric calibration of ERS satellite SAR images. IEEE Trans. Geosci. Remote Sens..

[4] Shimada M., Isoguchi O., Tadono T., Isono K. (2009). PALSAR Radiometric and Geometric Calibration. IEEE Trans. Geosci. Remote Sens..

[5] Covello F., Battazza F., Coletta A., Lopinto E., Fiorentino C., Pietranera L., Valentini G., Zoffoli S. (2010). COSMO-SkyMed an existing opportunity for observing the Earth. J. Geodyn..

[6] Luscombe A. (2010). Image quality and calibration of RADARSAT-2. Proceedings of the 2009 IEEE International Geoscience and Remote Sensing Symposium, IGARSS.

[7] Luscombe A.P. (2004). RADARSAT-2 SAR image quality and calibration operations. Can. J. Remote Sens..

[8] Schwerdt M., Bräutigam B., Bachmann M., Döring B. Efficient calibration and first results of TerraSAR-X. Proceedings of the Advanced SAR Workshop (ASAR).

[9] Schwerdt M., Bräutigam B., Bachmann M., Döring B., Schrank D., Gonzalez J.H. (2010). Final TerraSAR-X calibration result based on novel efficient methods. IEEE Trans. Geosci. Remote Sens..

[10] Schubert A., Small D., Miranda N., Geudtner D., Meier E. (2015). Sentinel-1A Product Geolocation Accuracy: Commissioning Phase Results. Remote Sens..

[11] Schwerdt M., Schmidt K., Tous-Ramon N., Castellanos-Alfonzo G., Doring B., Zink M., Prats P. (2016). Independent Verification of the Sentinel-1A System Calibration. IEEE J. Sel. Top. Appl. Earth Obs. Remote Sens..

[12] Eineder M., Minet C., Steigenberger P., Cong X., Fritz T. (2010). Imaging Geodesy—Towards Centimeter Level Ranging Accuracy With TerraSAR-X. IEEE Trans. Geosci. Remote Sens..

[13] Zhao R., Zhang G., Deng M. (2017). Multimode Hybrid Geometric Calibration of Spaceborne SAR Considering Atmospheric Propagation Delay. Remote Sens..

[14] Xu K., Jiang Y., Zhang G., Zhang Q., Wang X. (2017). Geometric Potential Assessment for ZY3-02 Triple Linear Array Imagery. Remote Sens..

[15] Liu X., Liu J., Hong W. (2006). The Analysis of the Precision in Space-borne SAR Image Location. J. Remote Sens..

[16] Zhao R., Zhang G., Deng M., Xu K., Guo F. (2017). Geometric Calibration and Accuracy Verification of the GF-3 Satellite. Sensors.

[17] Bräutigam B., Schwerdt M., Bachmann M. The External Calibration of TerraSAR-X, a Multiple Mode SAR-System. Proceedings of the European Conference on Synthetic Aperture Radar, DLR.

[18] Michael J., Donat P., David S., Schubert A., Meier E. (2008). Estimation of Atmospheric Path Delays in TerraSAR-X Data using Models vs. Measurements. Sensors.

[19] Schubert A., Jehle M., Small D., Meier E. (2010). Influence of Atmospheric Path Delay on the Absolute Geolocation Accuracy of TerraSAR-X High-Resolution Products. IEEE Trans. Geosci. Remote Sens..

[20] Breit H., Fritz T., Balss U., Lachaise M., Niedermeier A., Vonavka M. (2010). TerraSAR-X SAR Processing and Products. IEEE Trans. Geosci. Remote Sens..

[21] Small D., Schubert A. (2008). Guide to ASAR Geocoding.

[22] Qiu X., Han C., Liu J. (2013). A method for spaceborne SAR geolocation based on continuously moving geometry. J. Radars.

[23] Eftekhari A., Saadatseresht M., Motagh M. (2013). A study on rational function model generation for TerraSAR-X imagery. Sensors.

[24] Zhang G., Fei W., Li Z., Zhu X.Y., Li D.R. (2010). Evaluation of the RPC model for spaceborne SAR imagery. Photogramm. Eng. Remote Sens..

[25] Hua J., Zhang G. Research on the methods of inner calibration of spaceborne SAR. Proceedings of the 2011 IEEE International Geoscience and Remote Sensing Symposium (IGARSS).

[26] Chen E. (2004). Study on Ortho-Rectification Methodology of Space-Borne Synthetic Aperture Radar Imagery.

